# Effect of Frying and Roasting Processes on the Oxidative Stability of Sunflower Seeds (*Helianthus annuus*) under Normal and Accelerated Storage Conditions

**DOI:** 10.3390/foods10050944

**Published:** 2021-04-26

**Authors:** Arantzazu Valdés García, Ana Beltrán Sanahuja, Ioannis K. Karabagias, Anastasia Badeka, Michael G. Kontominas, María Carmen Garrigós

**Affiliations:** 1 Department of Analytical Chemistry, University of Alicante, Nutrition & Food Sciences, San Vicente del Raspeig, ES-03690 Alicante, Spain; ana.beltran@ua.es (A.B.S.); mc.garrigos@ua.es (M.C.G.); 2 Laboratory of Food Chemistry and Technology, Department of Chemistry, University of Ioannina, 45110 Ioannina, Greece; ikaraba@uoi.gr (I.K.K.); abadeka@cc.uoi.gr (A.B.); mkontomi@cc.uoi.gr (M.G.K.)

**Keywords:** sunflower seeds, frying, roasting, oxidative stability, HS-SPME-GC-MS, DSC, TGA, ATR-FTIR

## Abstract

The effect of different cooking processes such as frying and roasting on the oxidative stability of sunflower seeds was evaluated under accelerated oxidation and normal storage conditions. The fatty acid composition by GC-MS showed a higher amount of linoleic acid in fried samples due to the replacement of the seed moisture by the frying oil. On the other hand, roasted samples presented a higher oleic acid content. DSC and TGA results showed some decrease in the thermal stability of sunflower seed samples, whereas PV and AV showed the formation of primary and secondary products, with increasing oxidation time. Roasted sunflower seeds showed seven main volatile compounds characteristic of the roasting process by HS-SPME-GC-MS: 2-pentylfuran, 2,3-dimethyl-pyrazine, methyl-pyrazine, 2-octanone, 2-ethyl-6-methylpyrazine, trimethyl-pyrazine, and trans,cis-2,4-decadienal, whereas fried samples showed six volatile characteristic compounds of the frying process: butanal, 2-methyl-butanal, 3-methyl-butanal, heptanal, 1-hexanol, and trans,trans-2,4-decadienal. The generation of hydroperoxides, their degradation, and the formation of secondary oxidation products were also investigated by ATR-FTIR analysis. The proposed methodologies in this work could be suitable for monitoring the quality and shelf-life of commercial processed sunflower seeds with storage time.

## 1. Introduction

The production of sunflower seeds in Europe has recorded an important increase from 20 M to 42 M tonnes between 2009 and 2019, representing 75.8% of the world production followed by Asia (11%), the Americas (9%), and Africa (4%) [1]. Consumer preferences for sunflower seeds vary among the different European countries, mostly salted, roasted, and in-shell sunflower seeds for direct consumption [2]. Sunflower seeds are valuable and nutritious foods with a high oil content (35–40%) and protein (18–21%), with excellent potential to be used in the production of food formulation products and edible oils [3]. In addition, their content in substantial amounts of unsaturated fatty acids (77–82%), such as linoleic (59.0–67.5%) and oleic acids (14.0–18.1%) [4], render them susceptible to oxidative degradation and rancidity, resulting in their sensorial and nutritional deterioration [5]. The extent of the changes produced in lipid components of seeds depends on several properties such as the degree of unsaturation of fatty acids linked with the presence of factors promoting oxidation, mainly high temperatures, the presence of metallic ions, light, and oxygen [6]. Oxidation processes may occur during the storage of samples, with rancidity beginning on the seed surface to where the oxidation promoter, oxygen, diffuses from the environment into the bulk of the seed. This process is very complex, occurring via a free radical chain mechanism. In this process, a peroxyl radical acts, abstracting hydrogen from an unsaturated fatty acid molecule with weak C-H bonds (H in α position with respect to a double bond) [7].

In addition, heat treatments such as roasting and frying are necessary during nut processing in order to improve their sensory quality, digestibility, and microbiological safety. These cooking processes enhance nuts’ sensory and palatability characteristics such as flavor, color, and texture. During the roasting process, samples are heated to temperatures of 130–180 °C from 10 to 60 min [8,9], resulting in some physical and chemical changes of the product, e.g., formation of furan compounds, dehydration, and non-enzymatic browning. Thus, lipid stability can be affected in the roasted sample [10]. Furthermore, changes in fatty acid composition in nuts such as walnut [11], hazelnut [12], and almond [13,14,15] can occur during the roasting process. However, the effects of roasting temperatures on the fatty acid profile and antioxidant activity of sunflower seeds have not been thoroughly investigated.

On the other hand, during the deep frying process, nuts and seeds are immersed in different frying oils at 160–200 °C in the presence of air [16]. In this case, water vapor is released from the food being fried, allowing a rapid heat transfer and dehydration of the food in a time period ranging from seconds to several minutes. Thus, a significant lipid exchange between food and the frying oil is expected from foods with high lipid content such as nuts, which can lead to changes in fatty acid composition as reported in the literature [17]. In typical industrial frying operations, the frying oil is continuously and repeatedly used at high temperatures, being often regularly replenished with fresh oil [18]. Chemical reactions can result as a consequence of the presence of moisture from the food in combination with continuous oxygen exposition and high temperatures. As a consequence, degradation of the frying oil and also the food have been reported for sunflower [18] and canola oils [19].

The oxidative stability of fat samples has been frequently evaluated by determining the fatty acid composition using gas chromatography [20,21,22]. Chemical indicators used to estimate primary and secondary oxidation products in foods include peroxide (PV) [10], thiobarbituric acid (TBA), and *p*-anisidine (AV) [23]. Thermal techniques such as thermogravimetric analysis (TGA) and differential scanning calorimetry (DSC) have been also applied for the assessment of the oxidative deterioration of fatty foods, since the thermal parameters obtained can be directly related to the chemical structure of the sample [24]. In addition, thermal processing conditions may significantly modify the volatile profile of nuts. For this purpose, head space-solid phase microextraction–gas chromatography-mass spectrometry (HS-SPME-GC-MS) has been used as a rapid, sensitive economic technique without the use of solvents for volatiles extraction from nuts [25,26]. Attenuated total reflectance-Fourier transform infrared spectroscopy (ATR-FTIR) has been also used in order to monitor the main structural changes as a consequence of the oxidative treatment in nuts [20].

Most of the previously reported studies on nuts have not considered the influence of the cooking process (roasting and frying) under normal and accelerated oxidation conditions. In addition, the volatile profile of roasted and fried sunflower seeds has not been extensively reported to date. Thus, the aim of this study was to study the effect of different cooking processes (roasting and frying) on the oxidative stability of sunflower seeds. Thus, two different thermal treatments have been studied: normal storage and accelerated oxidation conditions. The fatty acid composition (GC-MS) was studied with primary and secondary oxidation products (PV and AV). Thermal parameters were obtained by using the DSC and TGA techniques. Additionally, the volatile profiles were determined by HS-SPME-GC-MS. Finally, lipids structural degradation was followed by ATR-FTIR. The methodologies used in this work based on DSC, TGA, HS-SPME-GC-MS, and ATR-FTIR techniques offer the advantage of performing a direct analysis of ground seeds compared to the analysis of the extracted seed/nut oil as it is reported for almond oil [26].

## 2. Materials and Methods

### 2.1. Chemicals

Sodium methylate (0.5 mol L^−1^ methanolic solution, HPLC grade), sulphuric acid (98%), acetic acid glacial (99.5%), sodium thiosulphate (0.1 mol L^−1^), starch solution, and n-hexane (99%, GC grade) were obtained from Panreac (Barcelona, Spain). Standards for GC analysis (99%) of methyl tridecanoate, oleic, linoleic, stearic, and palmitic acid methyl esters, petroleum ether (≥95%), methanol (≥99.8%), potassium iodide, chloroform (≥99.8%, for HPLC), acetic acid, hydrochloric acid, isooctane, potassium iodate, p-anisidine reagent, internal standard 4-methyl-2-pentanone, and hexanal (98%) were acquired from Sigma-Aldrich Inc. (St. Louis, MO, USA). All chemicals used were of analytical grade.

### 2.2. Materials

The commercially packaged sunflower seeds used in this study were obtained from a local market in Alicante (Spain). Roasted unshelled sunflower seeds with salt and shelled fried sunflower seeds with salt samples were analyzed. According to the package labeling, sunflower seed oil was used as the frying medium for shelled fried sunflower seeds. All samples were kept in a cool and dry place (storage temperature of 23 ± 1 °C and relative humidity of 50%). Samples were immediately prepared before analysis in order to protect them against oxidation. The shell of roasted sunflower seeds was removed by using a hammer, and 10 ± 1 g of nut seeds were ground in a domestic electric grinder (Moulinex, Barcelona, Spain). Then, the seed fragments were passed through a 1 mm sieve in order to ensure the homogeneity of the samples. Finally, samples were stored in a desiccator until analysis. Sunflower seed samples were directly analyzed by ATR-FTIR, HS-SPME-GC-MS, DSC, and TGA. For fatty acid composition and PV and AV determinations, the oil of samples was extracted by applying an analytical method previously developed [27]. Triplicates were carried out for all the analyses.

### 2.3. Oxidation Conditions

For accelerated oxidation conditions, sunflower seeds (1.0 ± 0.1 g) and extracted sunflower oil (12.0 ± 0.1 g) samples were placed into 20 mL dark glass vials sealed with an aluminium crimp cap provided with a polytetrafluoroethylene/silicone septum. In order to accelerate the oxidation, sample vials were placed in an oven (Selecta, Barcelona, Spain) at 100°. Four points of oxidative stability of samples were evaluated: zero, three, five, and 10 days of heat treatment. All the analyses were performed in triplicate. Samples subjected to normal storage conditions were kept protected from light into their commercial package made of polyethylene terephthalate/low density polyethylene at room temperature (25 ± 3 °C) for four and 11 months. Triplicate were carried out for all the analyses.

### 2.4. Analysis of FAMEs

The fatty acid composition of extracted sunflower oils was determined by GC-MS. FAMEs were prepared according to the American Oil Chemists’ Society method CE-2-66 [28], previously reported [20]. The fatty acid profile was analyzed using an Agilent 6890N gas chromatograph coupled to a 5973N quadrupole mass spectrometer operating in electronic impact (EI) ionization mode (70 eV). A BPX70 capillary column (30 m × 0.25 mm i.d. × 0.25 µm film thickness; SGE Europe Ltd., UK) was used. The column temperature was programmed from 120 to 245 °C (hold 15 min) at a heating rate of 3 °C min^−1^ using helium as carrier gas at a flow rate of 1 mL min^−1^. Ion source and GC-MS transfer line temperatures were kept at 250 and 280 °C, respectively. Injector temperature was fixed at 250 °C, and 1 µL of extracts were injected in the split mode (1:75). Prior to calibration curve runs, two different multistock solutions were mixed in n-hexane, consisting of 7000 mg Kg^−1^ of oleic and linoleic standards and a 1500 mg Kg^−1^ solution of stearic and palmitic acid methyl esters. Both were stored at −21 °C in amber glass vials. On each day of analysis, different concentrations of standard solutions were serially diluted. Identification of FAMEs in extracted sunflower oils was performed in full scan mode (*m/z* 30–550) by a combination of NIST mass spectral library and gas chromatographic retention times of standard compounds (oleic, linoleic, stearic, and palmitic acid methyl esters). A 700 mg Kg^−1^ solution of methyl tridecanoate was used as internal standard, as this fatty acid is not present in sunflower seeds.

### 2.5. Determination of Peroxide and p-Anisidine Values

The PV of all samples was determined according to ISO 3960:2007 Standard [29]. The secondary oxidation products formed were estimated by the determination of AV according to the IUPAC method 2.504 (IUPAC, 1987) [30].

### 2.6. Thermal Analysis

Ground seed samples analyzed by DSC and TGA in inert atmosphere (nitrogen). TA Instruments DSC Q2000 V23.12 Build 103 (New Castle, DE, USA) was used to carried out the DSC tests. TGA/SDTA 851 Mettler Toledo (Schwarzenbach, Switzerland) thermobalance was used to carried out the dynamic TGA tests; 3.0 ± 0.1 mg and 7.0 ± 0.1 mg of nut samples were used for DSC and TGA, respectively. Details of the methods used are reported elsewhere [31].

### 2.7. ATR–FTIR Analysis

The structural analysis of ground samples (2.00 ± 0.01 mg) was conducted using a Bruker Analitik IFS 66 FTIR spectrometer (Ettlingen, Germany) equipped with an ATR accessory (incident angle of 45°). Absorbance mode was recorded from 4.000–600 cm^−1^, using 64 scans and 4 cm^−1^ resolution. Correction against the background spectrum of air was carried out. All analyses were performed in duplicate.

### 2.8. HS–SPME–GC–MS Analysis

Ground samples (1.00 ± 0.01 g) with 2 mL of NaCl 2 mol L^−1^, 40 mL of the internal standard 4-methyl-2-pentanone (8 mg Kg^−1^), and a micro-stirring bar were placed in a 20 mL vial. SPME sampling and GC-MS determination were carried out as it is described in our previous work [31].

Hexanal was quantified by HS-SPME-GC-MS using a standard calibration curve as described elsewhere [32,33]. Stock (30 mg Kg^−1^) and working solutions were prepared using distilled water as solvent. The SPME sampling and GC-MS procedures were carried out as described before.

### 2.9. Statistical Analysis

SPSS software (Version 15.0, Chicago, IL, USA) was used to applied ANOVA to the results followed by the Tukey’s test. Significance of differences was considered at the level of *p* < 0.05.

## 3. Results and Discussion

### 3.1. Quantitative Analysis of FAMEs

The method used for FAMEs quantification was validated in terms of linearity, precision, detection (LOD), and quantification (LOQ) limits (Table 1). Linearity was estimated at five calibration concentration levels, each injected in triplicate. The calibration curves showed excellent linearity with R^2^ values above 0.993 and RSD values of peak areas lower than 5%. LOD and LOQ values for FAMEs of sunflower seeds ranged from 0.03–0.29 mg Kg^−1^ (3 S_y/x_/a) and 0.07–0.62 mg Kg^−1^ (10 S_y/x_/a), respectively, calculated by using regression parameters of the calibration graphs (where S_y/x_ is the standard deviation of the residues and a is the slope).

The concentrations (g fatty acid/100 g seed oil) of palmitic, stearic, oleic, and linoleic acids as a function of storage time at 100 °C (zero, three, five, and 10 days) and room temperature (four, eight, and 11 months) are shown in Table 2. After the accelerated oxidation treatment at 100 °C, the major fatty acid found in roasted seed oils at day 0 was oleic acid (63.35 ± 0.16%) followed by linoleic acid (30.92 ± 0.25%). There were also smaller amounts of palmitic (3.25 ± 0.06%) and stearic (2.38 ± 0.07%) acids. Similar results were reported for sunflower seeds [34] with 5.00–6.10% of palmitic, 2.53–3.24% of stearic, 54.1–54.1% of oleic, and 32.3–41.0% of linoleic fatty acids. Additionally, other seeds such as hemp, pumpkin, brown flaxseed, golden flaxseed, blue poppy seed, and white poppy seed oils have shown similar fatty acid composition [35,36,37]. Regarding fried samples, linoleic acid (77.40 ± 0.02%) was the highest fatty acid at the beginning of the storage period followed by oleic (14.31 ± 0.02%), palmitic (4.56 ± 0.19%), and, finally, stearic (3.73 ± 0.15%) acids. The higher value of linoleic content observed in fried sunflower seed samples compared to the roasted ones was related to sunflower seed processing. According to the package labeling, sunflower seed oil was used as the frying medium for these samples, which contains oleic (35%) and linoleic (54%) major fatty acids in its composition, as it has been reported [38]. In this sense, when the food is immersed in the oil during the frying process, partial migration of oil frying is produced into the samples. Additionally, some of the moisture of the food sample is replaced by the frying oil leading to the formation of pores allowing oil penetration into the created voids [31]. In this sense, changes in the composition of several nuts such as peanuts, almonds, cashew nuts, and sunflower seeds as a consequence of frying were reported showing a significant loss of moisture and oil gain, which were dependent on the structure of the samples [17]. This effect was more evident if combined with a high surface-to-weight ratio of sunflower seeds, showing an increase in their oil weight in relation to the moisture loss. A general increase in fatty acids concentration was observed with storage time at 100 °C except for linoleic acid content (Table 2), which decreased from 30.92 ± 0.25 to 25.46 ± 0.10 and from 77.40 ± 0.02 to 65.01 ± 2.61 g fatty acid/100 g seed oil for roasted and fried samples, respectively. This fact could be explained because the breakdown of polyunsaturated fatty acids, which allows the increase of unsaturated and saturated ones. Similar results were obtained for fatty acid composition of pumpkin seed and safflower oils after eight weeks of storage at 40 °C [36]. Similarly, Anjum et al. found that the longer the microwave roasting time (5, 10 and 15 min), the higher the percentage of oleic acid and the lower the linoleic acid content in sunflower seeds [39]. The increase in oleic acid and parallel decrease in linoleic acid contents observed with increasing oxidation time was attributed to the reported higher rate of fatty acid oxidation with increasing the number of double bonds. This fact is explained, since hydrogen attached to the carbon between two double bonds can be removed easily [20,40].

On the other hand, significant changes in the fatty acid composition of sunflower seeds were found with increasing oxidation time at room temperature (Table 2). In general, saturated fatty acids and oleic acid increased while the amount of linoleic acid decreased. Similar results were reported for the fatty acid composition of safflower oils after eight weeks of storage at 20 °C [36]. The decrease in linolenic acid with time was related to an increase in malondialdehyde content as oxidation proceeds, as previously reported [32].

### 3.2. Oxidative Indices: PV and AV

PV and AV values suggested that purchased samples were initially oxidized. Regarding PV, a similar behavior was observed for roasted and fried samples, presenting a high initial PV value (47 ± 1 and 41 ± 1 meq O_2_ kg^−1^ sunflower seed oil, respectively, Table 3), which may be explained by their high linoleic fatty acid content, being highly susceptible to oxidation during processing conditions. These results are in accordance with those reported in other studies for different vegetable oils, including almond and olive oils [41,42]. Under accelerated conditions (100 °C), PV analysis was conducted only at day 0, as no change in color during the titration of samples, corresponding to the formation of peroxides, was observed for three, five, and 10 days of oxidative treatment. Thus, it can be assumed that sunflower seeds were highly oxidized at this point of the study, due to their initial thermal processing. In this sense, the decomposition of hydroperoxides to secondary compounds was so fast that their presence would not be expected in samples at three, five, and 10 days of accelerated oxidative treatment. Similar results were reported for almonds during roasting [23], showing that hydroperoxide formation exhibits an initial formation followed by a degradation rates.

Regarding changes in AV (Table 3), similar values were observed for fried and roasted samples at day 0, detecting a rapid initial increase between day 0 and day 3 from 7 ± 1 to 46 ± 4 for roasted samples and from 8 ± 1 to 60 ± 7 for the fried samples, respectively. After that, no statistical differences (*p* > 0.05) were observed for roasted samples between five and 10 days; whereas AV of fried samples slightly decreased to 41 ± 3 at five days and, finally, AV increased to 53 ± 4 at the end of the thermal treatment (10 days). In general, AV tended to increase at 100 °C showing the formation of secondary oxidation products in accordance with the obtained PV values. Similar results were obtained in previous works with other nuts such as Spanish and American almond oils [27].

In order to effectively monitor the lipid oxidation process, the simultaneous detection of primary and secondary lipid oxidation products is necessary. Different results were observed for PV and AV values under accelerated oxidation treatment at 100 °C compared to those obtained during storage at room temperature. PV values at room temperature were determined at four and 11 months, expecting less oxidation of samples at room temperature compared to that at 100 °C. Regarding roasted samples, an increase (*p* < 0.05) in PV up to four months (77 ± 1 meq O_2_ kg^−1^ seed oil) and, finally, a decrease (*p* < 0.05) to 70 ± 2 meq O_2_ kg^−1^ seed oil after 11 months were obtained. As underlined by Poiana [43], the measurement of primary oxidation products is difficult, because they show a transitory nature. Then, PV increases only when the rate of peroxides formation exceeds that of its destruction. Thus, it is expected that samples were highly oxidized after 11 months of treatment, with a reduction in primary oxidation products, whereas the AV values obtained were higher after 11 months compared to four months due to the formation of secondary oxidations products. Regarding fried samples, an average increase (*p* < 0.05) from zero days to four months was found followed by a steady state up to 11 months, the PV values being higher for fried samples compared to the roasted ones. This behavior can be related to the high content of linoleic acid present in the fried samples after processing. For AV values, a similar behavior was observed for all the studied samples, indicating a simultaneous formation of secondary oxidation compounds and hydroperoxides.

In the present work, all samples were protected from light. Then, it was considered that the main route of sample degradation was the autoxidation. Despite the degradation mechanism, temperature and oxygen concentration are the main external variables that determine the rates of formation of lipid peroxy radicals and hydroperoxides. According to a previous work [44], under normal degradation treatment with moderate temperature, such as room temperature, the solubilization of oxygen is high. As a result, the oxidation process is initiated, and lipid alkyl radicals are the most common species present and hydroperoxides are the major products formed. However, when temperatures are high, as in the accelerated treatment at 100 °C, the degradation is more complex, because two reactions are involved simultaneously: oxidative and thermal reactions. In this case, the solubilization of oxygen decreases, which allows the increase of alkyl radicals concentration and higher decomposition of hydroperoxides that allows the formation of new compounds.

### 3.3. Thermal Analysis

**Figure 1:**
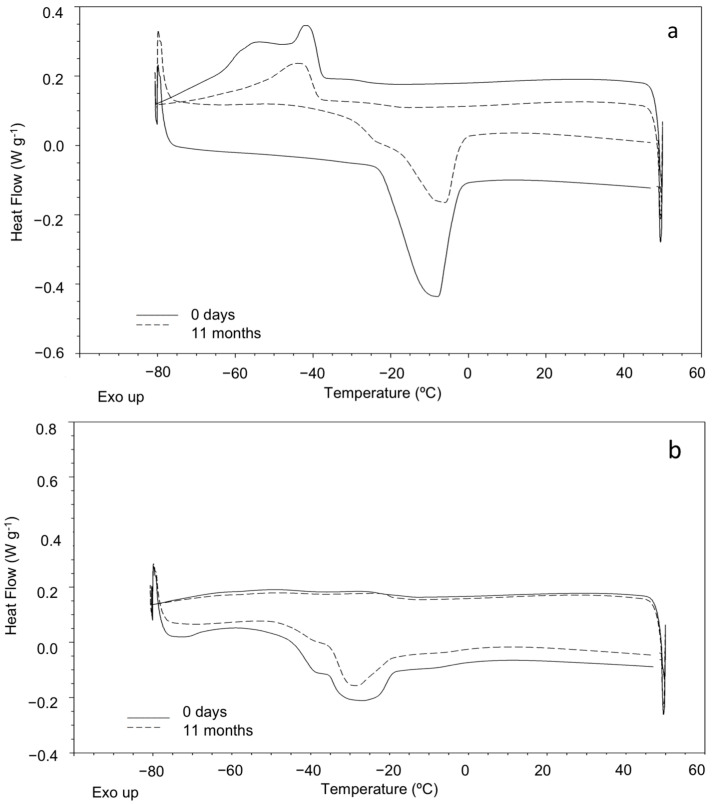
DSC curves obtained for roasted (**a**) and fried (**b**) sunflower seed samples at zero days and 11 months of storage at room temperature.

The effect of oxidative conditions on the thermal stability of fried and roasted sunflower samples was studied by DSC and TGA. The DSC curves obtained for roasted and fried samples at zero days and 11 months of storage at room temperature (Figure 1) showed an exothermic crystallization over a temperature range, which was related to the polymerization of different constituents of the samples. Additionally, samples showed an endothermic melting thermal transition, which corresponds to the thermal decomposition of the fatty acids. Fried samples did not show any exothermic transition at day 0, but a melting transition consisting of complex overlapped peaks. The DSC cooling curve observed for fried sunflower seed samples was similar to the cooling thermal profile of sunflower vegetable oil, as reported in a previous study [45]. These results are in accordance with those obtained from the fatty acid composition by GC-MS in the present study, which supported that the frying process changes the composition of the samples due to frying oil penetration into the product, resulting in different structural and thermal properties for fried sunflower seed samples. Four DSC parameters were determined: crystallization temperature: T_c_ (°C); melting temperature: T_m_ (°C); crystallization enthalpy: ∆H_c_ (J g^−1^); and melting enthalpy: ∆H_m_ (J g^−1^) (Table 4). In general, as oxidation time increased, the peaks corresponding to crystallization and melting transitions totally disappeared for all the studied samples.

**Figure 2:**
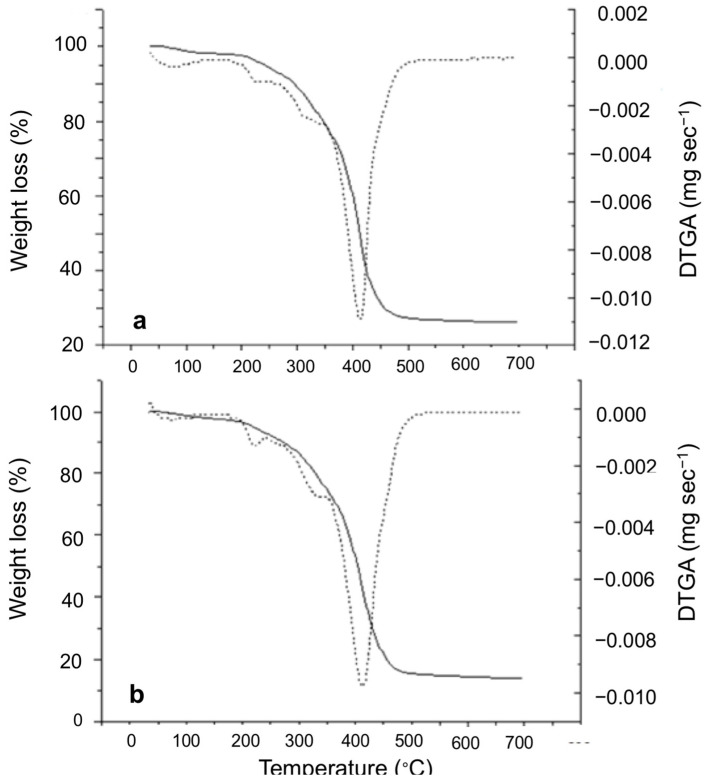
Thermogravimetric (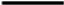) profiles and first derivative curves (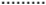) at day 0 obtained for roasted (**a**) and fried (**b**) sunflower seed samples.

Similar thermal degradation profiles for all samples at day 0 of the oxidative treatment were obtained by TGA. Figure 2 shows the TGA curves obtained for roasted (Figure 2a) and fried (Figure 2b) sunflower seeds at day 0 of the oxidative treatment, where a profile of sample weight loss with temperature occurring at four different stages was obtained. An initial step was observed between 30–140 °C as a result of the loss of volatile compounds and water evaporation [46,47]. The two following overlapped thermal decomposition steps (170–360 °C) were attributed to the thermooxidative degradation of the complex sample matrix. Finally, the main degradation step, with maximum weight loss around 400 °C, represents the rupture of covalent bonding and changes in the chemical structure [45].

In order to monitor the thermal stability of sunflower seed samples under different oxidative treatments, two TGA parameters were determined: weight loss (%) and maximum degradation temperature, T_max_ (°C) (Table 4). As expected, roasted samples showed a higher initial weight loss of 60% compared to the fried ones (53%) due to their initial thermal processing. In principle, roasting always involves the dehydration with moisture removal during processing and microstructure changes with a porosity increase, which leads to the migration of storage oil from cell to cell within the food matrix. Thus, the loss of moisture and microstructure porosity could be related to the loss of overall nut weight [48]. In fried samples, no significant differences (*p* > 0.05) were observed in weight loss (%) during the oxidative stability study at 100 °C, which could be attributed to the deep frying process. In this sense, the moisture loss from the food is partially replaced by oil absorbed from the frying medium by the food [49]. Regarding T_max_, similar values were obtained at day 0 for roasted and fried samples. However, after 10 days of oxidative treatment at 100 °C, significant differences in thermal stability were observed. In this sense, roasted samples were less stable as T_max_ decreased from 414 ± 1 to 403 ± 1 °C, followed by the fried ones with a T_max_ decrease from 416 ± 1 to 407 ± 1 °C. Some authors suggested that transferred oil during the deep frying process may carry minor components, such as bioactive compounds, from the oil used as frying medium that would expectedly enhance the oxidative stability of the prepared food [49]. In particular, the sunflower seed oil used as the frying medium in this study has been reported to show noticeable amounts of tocopherols [50,51]. On the other hand, no significant differences (*p* > 0.05) in T_max_ and weight loss (%) values were obtained for samples during treatments.

### 3.4. Effect of Oxidative Conditions on Volatiles Profile Composition

#### 3.4.1. Volatiles Profile Determination at the Beginning of the Oxidative Treatment

**Figure 3:**
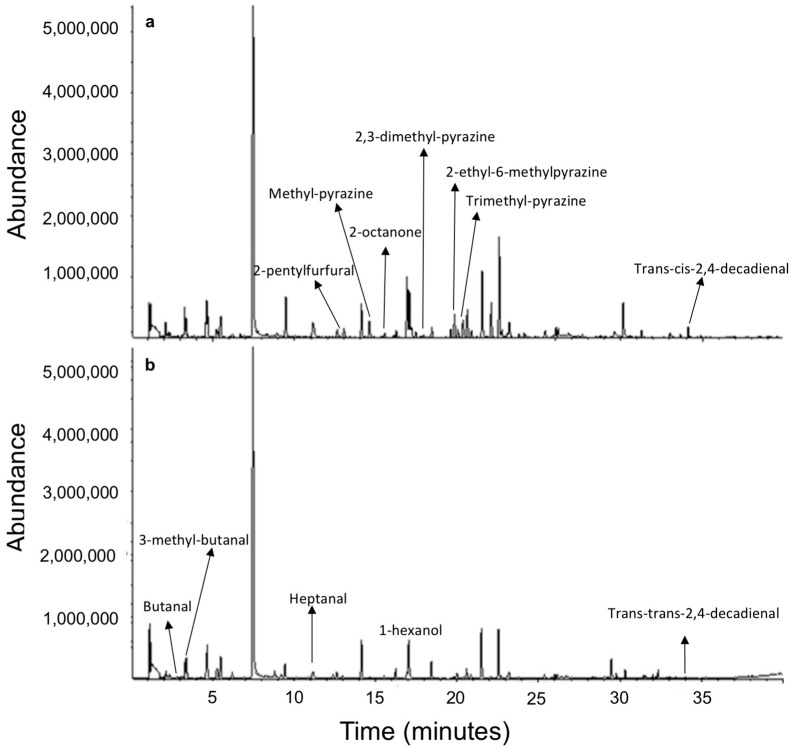
Full-scan chromatograms obtained for roasted (**a**) and fried (**b**) sunflower seed samples at day 0 of oxidative treatment.

Roasted and fried sunflower seeds showed significant differences regarding main volatile compounds detected at the beginning of the oxidative treatment. A total of 25 volatile common compounds were identified for roasted and fried sunflower seed samples at day 0 (Table 5). However, roasted sunflower seeds showed seven additional compounds that are characteristic of the roasting process (Figure 3a), such as 2-pentylfuran, 2,3-dimethyl-pyrazine, methyl-pyrazine, 2-octanone, 2-ethyl-6-methylpyrazine, trimethyl-pyrazine, and trans,cis-2,4-decadienal. Regarding fried samples, they showed five additional compounds being characteristic of the deep frying process (Figure 3b): butanal, 3-methyl-butanal, heptanal, 1-hexanol, and trans,trans-2,4-decadienal. In general, the volatile profile of the analyzed sunflower samples showed a large variety of terpenic compounds that are characteristic constituents of the volatile fraction of the sunflower seed oil [31].

As it was expected, several aldehydes, which are derivatives from the lipid oxidation, were found. Hexanal, heptanal, and nonanal, derived from the oxidation of linoleic acid [33], were detected in the analyzed samples. This fact can be explained due to the high linoleic fatty acid content of sunflower seeds and also due to their high surface-to-weight ratio, showing an increase in their fatty acid decomposition and thermal degradation when sunflower seeds are processed at high temperatures. Regarding the oxidation of linoleic acid, trans-2-heptenal is related to the rancid odour of nuts [52]. Additionally, higher amounts of volatile compounds for roasted sunflower seeds were obtained at day 0, in contrast to the fried ones that can be related to the different initial thermal processes of both samples. According to the literature, frying is carried out at 160–200 °C over a period of some minutes (2–5 minutes), while during the roasting process, samples are heated to a temperature of 130–180 °C from 10 to 60 min [8,16,53,54]. Roasting carried out during longer times allows the characteristic roasted flavor due to Maillard reactions and the Strecker degradation of amino acids [48]. On the other hand, during the deep-frying process, water vapor is released from the food being fried, allowing a rapid heat transfer and dehydration of food in a time period ranging from seconds to several minutes. Thus, a significant lipid exchange between seeds and the frying oil is expected, which affects the volatile profile [17].

#### 3.4.2. Volatile Profile of Sunflower Seed Samples with Oxidation Time at 100 °C

During the oxidative treatment, some characteristic initial volatiles disappeared, whereas new ones were subsequently obtained. Regarding roasted samples, only 20 of the 32 volatile compounds identified on day 0 (pentanal, alpha-pinene, hexanal, 2-heptanone, trans-2-hexenal, 2-pentylfuran, 1-pentanol, methyl-pyrazine, 2-octanone, octanal, 2,5-dimethylpyrazine, trans-2-heptenal, 2-ethyl-6-methylpyrazine, 2-ethyl-5-methylpyrazine, nonanal, 3-octen-2-one, 3-ethyl-2,5-dimethyl-pyrazine, 1-octen-3-ol, furfural, and benzaldehyde) were observed until the end of the oxidative treatment (day 10). Regarding fried samples, only 13 of the 30 volatile compounds identified on day 0 (2-methyl-butanal, pentanal, hexanal, 2-heptanone, 1-pentanol, octanal, trans-2-heptenal, nonanal, 3-octen-2-one, 1-octen-3-ol, furfural, benzaldehyde, and trans, trans-2,4-decadienal) were observed until the end of the oxidative treatment (day 10).

According to the information provided on the commercial packaging material, sunflower seed oil was used for frying the studied samples; thus, volatile products derived from linoleic acid, which is the main fatty acid present in sunflower seed oil, were observed in large amounts for fried sunflower seeds during the oxidative treatment at 100 °C: nonanal, heptanal, and trans-2-heptenal. Additionally, it is interesting to note the behavior observed from the third day until the end of the oxidative treatment, where two additional volatile compounds derived from linoleic acid oxidation were found, 1-octen-3-ol and 2-pentylfurfural, which were present in higher quantities in fried samples compared to the roasted ones. The presence of trans, trans-2,4-decadienal in roasted and fried samples is also indicative of sunflower seeds oxidation [31]. On the other hand, the concentration of 1-pentanol increased as a linoleic acid oxidation product up to day 5, when there was a final decrease until the end of the oxidative treatment at a rate proportional to the amount of oxygen present in the headspace vial [31].

#### 3.4.3. Hexanal Determination by HS-SPME-GC-MS

Hexanal is a linoleic acid breakdown product that is recognized as a good marker of rancidity, being directly related to the development of oxidative off-flavors [55]. As it can be seen in Table 5, the initial content of hexanal (day 0) was slightly higher in roasted seed samples compared to the fried ones, this behavior being in accordance with previous studies reported in almond samples [31]. The decrease in the content of this aldehyde from zero to three days of thermal treatment may be due to the reaction between oxygen and unsaturated fatty acids of the sample and the depletion of headspace oxygen in the closed vial [26]. As the oxidative treatment progresses, an increase in hexanal content was observed between three and 10 days for roasted and fried sunflower seed samples as a consequence of samples oxidation. At the end of the accelerated oxidation treatment, fried samples showed slightly lower (*p* < 0.05) values of hexanal content compared to roasted ones. According to some authors, tissue breakdown during the roasting process is a major factor controlling lipid stability in roasted products such as peanuts, almonds, or hazelnuts [48]. The loss of compartmentalization and the increase in porosity accelerate mass transfer, resulting in facilitating the access of oxygen into the food tissues.

### 3.5. ATR-FTIR Analysis

**Figure 4:**
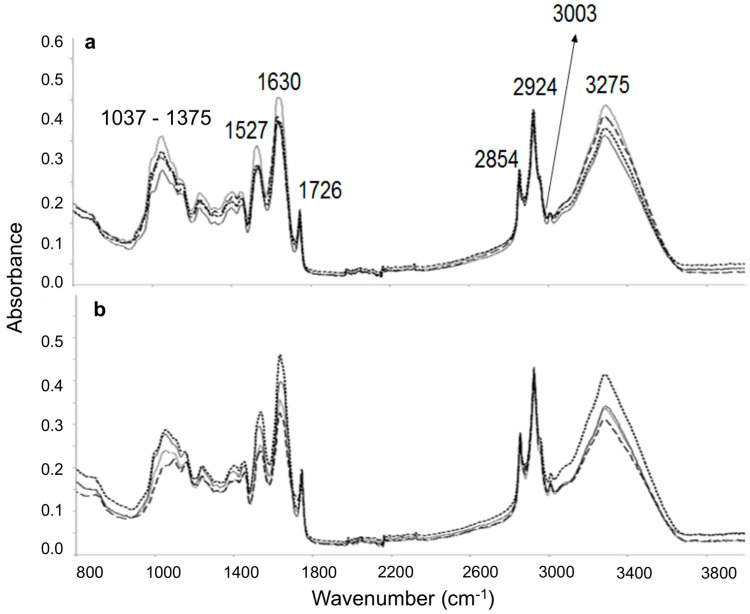
ATR-FTIR spectra of roasted (**a**) and fried (**b**) sunflower seed samples at zero (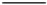), three (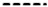), five (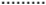), and 10 (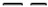) days of oxidative treatment at 100 °C.

**Figure 5:**
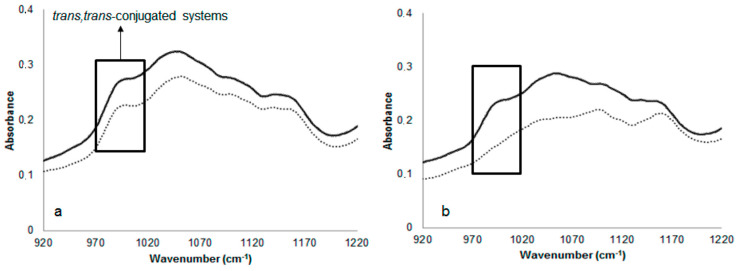
ATR-FTIR spectra zoomed region at 1220–920 cm^−1^ of fried (**a**) and roasted (**b**) sunflower seed samples at zero (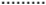) and 10 (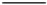) days of oxidative treatment at 100 °C.

Figure 4 shows the typical absorption bands of sunflower seed samples under oxidative conditions at 100 °C obtained by ATR-FTIR. According to previous works, the FTIR bands assigned in sunflower seed oils and the changes obtained throughout the oxidation process showed differences among samples [56]. A similar behavior was observed in the present study, where three different stages were distinguished in the oxidation process: the generation of hydroperoxides followed by their degradation and the formation of secondary oxidation products [31]. In this sense, the main changes appeared in the following different regions:

(a) Region 3700–3010 cm^−1^: The band near 3275 cm^−1^ intensifies as the oxidation degree of the samples increases. This fact could be related to hydroperoxides [17]. As the oxidation process advances, the degradation of these primary products and the formation of secondary oxidation products resulted in the decrease of the intensity of this band.

(b) Region 3010–2990 cm^−1^: The band at approximately 3003 cm^−1^ is associated to the stretching vibration of CH cis-olefinic groups. Double bounds of unsaturated fatty acids underwent cis to trans isomerization with the oxidation time. Thus, the gradual disappearance of this band was observed for all samples [16].

(c) Region 2955–2800 cm^−1^: In general, an increase in the intensities of the absorption bands at 2924 and 2854 cm^−1^, which were related to CH_2_ asymmetric and symmetric stretching vibrations, respectively, were observed for all samples.

(d) Bands at 1726, 1630, and 1527 cm^−1^ associated to the presence of CH bending vibrations in CH_2_ and CH_3_ increased their intensities due to the oxidative treatment in roasted and fried samples.

(e) Region 988–955 cm^−1^: This region was associated to the trans, trans-conjugated dienic systems absorption (near 988 cm^−1^), and it could provide information on the gradual cis to trans isomerization and the oxidative state of the samples throughout the oxidative treatment. As it can be seen in Figure 5, some differences were observed as oxidation time increased at 100 °C. In this sense, this band was evident at day 0 for both samples, being more evident at the end of the oxidative treatment (10 days) for fried seeds compared to roasted sunflower seed samples. These findings are in accordance with previous results obtained for volatiles, as large amounts of trans, trans 2,4 decadienal, trans, 2-heptenal, and trans, 2-hexenal were found in fried sunflower seed samples (Table 5).

## 4. Conclusions

Roasting and deep frying are different thermal processes carried out by the food industry in order to improve the properties of food, namely, flavor and crispness characteristics. As a consequence of this specific processing, roasted and fried sunflower seeds are susceptible to oxidation and structural changes due to their specific composition. This study underlies the suitability of the proposed methods to show the impact of roasting and frying treatments on the chemical composition of sunflower seeds. Indeed, all the studied parameters could be related to the oxidative damage of the tested samples during accelerated oxidation and normal storage conditions. The obtained results showed a different initial composition regarding the fatty acids content of samples. As a consequence, roasted and fried samples were oxidized in a different manner under 100 °C and storage at ambient temperature. The processing treatment and oxidative stability of sunflower seeds is important to ensure their nutritional quality and, therefore, their health benefits offered by the monounsaturated and polyunsaturated fatty acid profiles, which could be related to a beneficial blood serum lipid profile and a lower incidence of cardiovascular diseases. Finally, it is important to ensure the proper processing and storage of sunflower seeds to maintain the organoleptic properties as well as the consumers’ acceptance.

## Figures and Tables

**Table 1:** Analytical figures of merit for fatty acids determination by GC-MS in sunflower seed samples.

Compound	R^2^	Linear Range (g Kg^−^^1^)	LOD (mg Kg^−^^1^)	LOQ (mg Kg^−^^1^)
Oleic acid (roasted seeds)	0.9928	3.00–7.00	0.29	0.50
Oleic acid (fried seeds)	0.9928	0.40–2.50	0.29	0.50
Linoleic acid	0.9979	2.50–5.50	0.19	0.62
Stearic acid	0.9995	0.10–1.00	0.07	0.17
Palmitic acid	0.9995	0.10–1.00	0.03	0.07

**Table 2:** Major fatty acid content of sunflower seeds (g fatty acid/100 g seed oil) as a function of storage time for accelerated and room temperature oxidation conditions.

Fatty Acid	Storage Treatment	Storage Time	Roasted Sample	Fried Sample
Palmitic	100 °C	0 days	3.25 ± 0.06 ^a^	4.56 ± 0.19 ^a^
		3 days	6.77 ± 0.23 ^b^	4.66 ± 0.29 ^a^
		5 days	6.19 ± 0.50 ^b^	4.64 ± 0.10 ^a^
		10 days	6.66 ± 0.75 ^b^	5.99 ± 0.27 ^b^
	Room temperature	0 days	3.25 ± 0.06 ^a^	4.56 ± 0.19 ^a^
		4 months	6.90 ± 0.10 ^c^	5.95 ± 0.09 ^b^
		11 months	6.09 ± 0.02 ^b^	8.47 ± 0.02 ^c^
Stearic	100 °C	0 days	2.38 ± 0.07 ^a^	3.73 ± 0.15 ^a^
		3 days	3.77 ± 0.57 ^b^	4.33 ± 0.50 ^ab^
		5 days	3.08 ± 0.24 ^ab^	4.91 ± 0.15 ^b^
		10 days	4.41 ± 0.21 ^c^	6.34 ± 0.42 ^c^
	Room temperature	0 days	2.38 ± 0.07 ^a^	3.73 ± 0.15 ^a^
		4 months	4.11 ± 0.02 ^b^	4.70 ± 0.01 ^b^
		11 months	4.29 ± 0.04 ^c^	6.58 ± 0.02 ^c^
Oleic	100 °C	0 days	63.35 ± 0.16 ^a^	14.31 ± 0.02 ^a^
		3 days	68.12 ± 0.07 ^c^	16.35 ± 0.88 ^a^
		5 days	67.25 ± 0.12 ^b^	19.06 ± 1.86 ^b^
		10 days	67.43 ± 0.03 ^b^	22.36 ± 0.10 ^c^
	Room temperature	0 days	63.35 ± 0.16 ^a^	14.31 ± 0.02 ^a^
		4 months	81.30 ± 0.20 ^b^	17.50 ± 0.03 ^b^
		11 months	82.31 ± 0.50 ^b^	22.70 ± 0.20 ^c^
Linoleic	100 °C	0 days	30.92 ± 0.25 ^a^	77.40 ± 0.02 ^a^
		3 days	26.16 ± 0.10 ^b^	73.77 ± 1.40 ^a^
		5 days	25.37 ± 0.72 ^b^	72.63 ± 2.22 ^a^
		10 days	25.46 ± 0.10 ^b^	65.01 ± 2.61 ^b^
	Room temperature	0 days	30.92 ± 0.25 ^a^	77.40 ± 0.02 ^a^
		4 months	7.72 ± 0.04 ^b^	71.80 ± 0.10 ^b^
		11 months	7.31 ± 0.03 ^c^	62.20 ± 0.20 ^c^

Mean ± SD, *n* = 3. Different superscripts for each fatty acid within the same column and storage treatment indicate statistically significant different values (*p* < 0.05).

**Table 3:** Changes in PV (meq O_2_ kg^−1^ seed oil) and AV (absorbance units at 350 nm g^−1^ seed oil) values for roasted and fried sunflower seed oils as a function of oxidation time at 100 °C and storage at room temperature.

Storage Treatment	Time	Roasted		Fried	
		PV	AV	PV	AV
100 °C	0 days	47 ± 1	7 ± 1 ^a^	41 ± 1	8 ± 1 ^a^
	3 days	nd	46 ± 4 ^b^	nd	60 ± 7 ^c^
	5 days	nd	40 ± 8 ^b^	nd	41 ± 3 ^b^
	10 days	nd	44 ± 4 ^b^	nd	53 ± 4 ^c^
Room temperature	0 days	47 ± 1 ^a^	7 ± 1 ^a^	41± 1 ^a^	8 ± 1 ^a^
	4 months	77 ± 1 ^c^	11 ± 2 ^b^	148 ± 3 ^b^	14 ± 3 ^b^
	11 months	70 ± 2 ^b^	17 ± 1 ^c^	141 ± 3 ^b^	17 ± 1 ^b^

Mean ± SD (*n* = 3). nd: non detected color changes during tritation of samples. Different superscripts for each fatty acid within the same column and storage treatment indicate statistically significant different values (*p* < 0.05).

**Table 4:** DSC and TGA parameters (mean value (*n*= 3) ± SD) obtained for sunflower seeds during the oxidative stability study at 100 °C and storage time at room temperature. Different superscripts for each sample within the same column and treatment indicate statistically significant different values (*p* < 0.05). nd: non detected.

Sample	Storage Treatment	Oxidation Time	T_c_ (°C)	ΔH_c_ (J g^−1^)	T_m_ (°C)	ΔH_m_ (J g^−1^)	T_max_ (°C)	Weight Loss (%) *
Roasted sunflower seed	100 °C	0 days	−47 ± 8	17 ± 1	−10 ± 2	27 ± 2	414 ± 1 ^a^	60 ± 3 ^a^
		3 days	nd	nd	nd	nd	407 ± 1 ^b^	52 ± 3 ^b^
		5 days	nd	nd	nd	nd	404 ± 1 ^c^	50 ± 4 ^b^
		10 days	nd	nd	nd	nd	403 ± 1 ^c^	50 ± 3 ^b^
	Room temperature	0 days	−47 ± 8 ^a^	17 ± 1 ^a^	−10 ± 2 ^a^	27 ± 2 ^a^	414 ± 1 ^a^	60 ± 3 ^a^
		4 months	−59 ± 1 ^b^	9 ± 1 ^b^	−12 ± 1 ^a^	23 ± 1 ^b^	412 ± 1 ^a^	56 ± 1 ^a^
		11 months	nd	nd	nd	nd	413 ± 1 ^a^	52 ± 3 ^a^
Fried sunflower seed	100 °C	0 days	nd	nd	−28 ± 1	34 ± 4	416 ± 1 ^a^	53 ± 3 ^a^
		3 days	nd	nd	nd	nd	404 ± 2 ^b^	53 ± 1 ^a^
		5 days	nd	nd	nd	nd	407 ± 1 ^b^	53 ± 4 ^a^
		10 days	nd	nd	nd	nd	407 ± 1 ^b^	53 ± 3 ^a^
	Room temperature	0 days	−47 ± 8	17 ± 1	−10 ± 2 ^a^	27 ± 2 ^b^	414 ± 1 ^a^	60 ± 3 ^a^
		4 months	nd	nd	−30 ± 1 ^b^	33 ± 1 ^a^	413 ± 2 ^a^	52 ± 1 ^a^
		11 months	nd	nd	−30 ± 2 ^b^	19 ± 2 ^c^	414 ± 1 ^a^	55 ± 2 ^a^

* Corresponding to the main degradation step.

**Table 5:** Volatile compounds (µg·Kg^−1^) identified in roasted and fried sunflower seeds under accelerated oxidative treatment at 100 °C (mean ± SD, *n* = 3).

			Oxidative Treatment (Days)							
			0		3		5		10	
Volatile Compound	t_R_ (min)	Q_f_	Roasted	Fried	Roasted	Fried	Roasted	Fried	Roasted	Fried
2-methyl-propanal	2.179	91	nd	nd	466.5 ± 58.7 ^a^	nd	133.7 ± 8.5 ^b^	787.9 ± 46.1	nd	nd
Butanal	2.857	91	nd	36.5 ± 2.9 ^a^	nd	nd	432.1 ± 32.0 ^a^	457.4 ± 82.2 ^b^	304.7 ± 22.9 ^b^	211.5 ± 62.6 ^c^
2-Butanone	3.179	91	nd	nd	608.9 ± 65.5 ^a^	nd	450.7 ± 38.2 ^b^	nd	158.9 ± 20.4 ^c^	nd
2-methyl-butanal	3.326	91	524.5 ± 64.7 ^a^	164.5 ± 2.9 ^a^	617.2 ± 39.5 ^a^	3395.9 ± 748.9 ^b^	330.1 ± 44.4 ^b^	1117.4 ± 66.1 ^c^	nd	85.5 ± 9.6 ^d^
3-methyl-butanal	3.319	95	nd	157.3 ± 11.1 ^a^	nd	1203.6 ± 201.6 ^b^	85.6 ± 7.2	256.0 ± 13.6 ^c^	nd	nd
1-chloro-pentane	3.847	91	nd	nd	nd	1664.4 ± 225.1 ^a^	nd	549.0 ± 77.4 ^b^	nd	114.3 ± 4.4 ^c^
Pentanal	4.654	91	1112.4 ± 132.2 ^a^	491.1 ± 35.4 ^a^	3650.8 ± 205.5 ^b^	2920.7 ± 416.8 ^b^	5021.1 ± 537.7 ^c^	6366.5 ± 416.9 ^c^	2846.5 ± 193.4 ^d^	2280.6 ± 571.3 ^b^
alpha-pinene	5.498	96	868.6 ± 18.8 ^a^	383.6 ± 50.9 ^a^	656.35 ± 50.3 ^b^	nd	334.3 ± 45.0 ^c^	902.2 ± 141.3 ^b^	152.8 ± 22.2 ^d^	258.5 ± 60.4 ^a^
Toluene	6.229	90	117.9 ± 25.9 ^a^	91.1 ± 4.5 ^a^	123.8 ± 24.2 ^b^	nd	143.6 ± 19.2 ^b^	203.0 ± 21.8 ^b^	nd	nd
Camphene	6.710	97	77.8 ± 13.2 ^a^	20.3 ± 0.8 ^a^	80.2 ± 5.8 ^a^	nd	83.1 ± 9.8 ^a^	90.3 ± 10.8 ^b^	nd	nd
Methyl-disulfide	7.189	96	nd	nd	nd	291.0 ± 40.0 ^a^	192.4 ± 9.2	218.1 ± 49.8 ^a^	nd	nd
Hexanal	7.490	95	5750.0 ± 25.0 ^a^	5383.1 ± 180.9 ^a^	4123.0 ± 216.2 ^b^	3766.7 ± 281.0 ^b^	4866.7 ± 325.3 ^b^	5600.0 ± 114.6 ^a^	5616.7 ± 350.2 ^a^	5658.3 ± 87.8 ^a^
beta-Pinene	7.793	92	170.1 ± 26.6	137.2 ± 20.9	nd	nd	nd	nd	nd	nd
Sabinene	8.268	95	162.0 ± 35.7	57.7 ± 5.9	nd	nd	nd	nd	nd	nd
2-n-butyl-furan	8.957	90	nd	nd	nd	nd	630.6 ± 152.2 ^a^	758.9 ± 107.5 ^a^	213.3 ± 57.0 ^b^	248.0 ± 30.9 ^b^
2-Heptanone	11.129	92	432.2 ± 53.1 ^a^	57.6 ± 5.9 ^a^	2631.9 ± 602.1 ^b^	3244.4 ± 754.4 ^b^	4349.5 ± 921.2 ^c^	5009.5 ± 907.0 ^c^	2921.7 ± 487.2 ^b^	1925.4 ± 99.6 ^d^
Heptanal	11.206	97	nd	111.6 ± 9.3 ^a^	nd	3965.8 ± 299.9 ^b^	1974.0 ± 332.7	3502.5 ± 470.5 ^b^	nd	nd
Trans-2-hexenal	11.634	97	231.7 ± 22.5 ^a^	90.3 ± 1.7 ^a^	5657.3 ± 421.1 ^b^	nd	484.1 ± 84.1 ^c^	2053.7 ± 289.9 ^b^	400.0 ± 41.5 ^c^	616.8 ± 19.6 ^c^
2-Pentylfuran	13.048	94	153.6 ± 19.9 ^a^	nd	464.2 ± 63.4 ^b^	25381.0 ± 812.6 ^a^	10365.5 ± 456.4 ^c^	23995.3 ± 30.3 ^b^	7574.4 ± 522.6 ^d^	12638.0 ± 511.3
1-Pentanol	14.107	90	835.8 ± 121.8 ^a^	423.4 ± 13.1 ^a^	604.5 ± 65.1 ^b^	5985.3 ± 761.4 ^b^	6361.8 ± 1055.0 ^c^	6988.7 ± 152.9 ^c^	2243.2 ± 251.3 ^d^	1268.8 ± 367.8 ^d^
Methyl-pyrazine	14.598	91	383.0 ± 47.4 ^a^	nd	9728.2 ± 932.3 ^b^	2152.4 ± 151.6 ^a^	1032.1 ± 103.6 ^c^	1603.7 ± 152.9 ^b^	727.0 ± 99.7 ^d^	308.9 ± 37.2 ^c^
2-Octanone	15.385	91	27.2 ± 7.6 ^a^	nd	1021.6 ± 154.8 ^b^	691.8 ± 152.7 ^a^	1226.1 ± 162.6 ^b^	nd	476.7 ± 45.0 ^c^	342.2 ± 57.8 ^b^
Octanal	15.547	94	123.4 ± 38.3 ^a^	33.5 ± 2.8 ^a^	897.6 ± 44.0 ^b^	3017.6 ± 970.7 ^b^	1222.8 ± 121.6 ^c^	1749.3 ± 98.6 ^c^	506.0 ± 63.3 ^d^	422.6 ± 57.6 ^d^
2,5-Dimethylpyrazine	16.895	91	1486.5 ± 56.1 ^a^	87.1 ± 6.6 ^a^	623.9 ± 53.9 ^b^	1654.6 ± 317.4 ^b^	1819.1 ± 88.7 ^c^	1274.6 ± 44.9 ^b^	670.4 ± 57.5 ^b^	nd
trans-2-Heptenal	17.045	93	1155.3 ± 285.9 ^a^	554.7 ± 8.1 ^a^	1975.0 ± 155.1 ^b^	498.0 ± 70.2 ^b^	4106.6 ± 609.3 ^c^	955.9 ± 122.0 ^c^	2322.1 ± 215.0 ^b^	3308.8 ± 192.1 ^d^
1-Hexanol	17.499	91	nd	207.5 ± 2.7	nd	nd	nd	nd	nd	nd
2,3-dimethyl-pyrazine	17.914	90	93.1 ± 11.3 ^a^	nd	100.3 ± 15.7 ^a^	221.1 ± 88.9	124.5 ± 22.4 ^a^	nd	nd	nd
Dimethyl-trisulfide	19.283	92	nd	nd	nd	388.0 ± 60.1	nd	nd	nd	nd
2-ethyl-6-methylpyrazine	19.572	91	178.8 ± 26.9 ^a^	nd	2890.0 ± 917.5 ^b^	1379.1 ± 195.5 ^a^	505.3 ± 86.5 ^c^	971.9 ± 69.7 ^b^	420.1 ± 82.8 ^c^	224.6 ± 48.3 ^c^
2-Ethyl-5-methylpyrazine	19.806	93	492.3 ± 70.7 ^a^	31.4 ± 3.7 ^a^	532.9 ± 90.5 ^a^	1231.6 ± 238.8 ^b^	1057.6 ± 193.5 ^b^	1006.8 ± 71.9 ^b^	473.5 ± 88.1 ^a^	nd
Nonanal	20.018	91	212.9 ± 86.5 ^a^	93.4 ± 8.8 ^a^	1079.4 ± 79.8 ^b^	7549.2 ± 570.1 ^b^	1296.8 ± 163.5 ^b^	1792.8 ± 119.1 ^c^	609.6 ± 67.0 ^c^	709.0 ± 88.9 ^d^
Trimethyl-pyrazine	20.323	91	521.3 ± 54.1 ^a^	nd	566.4 ± 65.5 ^a^	nd	889.5 ± 49.0 ^b^	nd	nd	nd
3-Octen-2-one	20.592	91	828.1 ± 181.4 ^a^	130.2 ± 1.6 ^a^	970.0 ± 29.2 ^a^	1756.5 ± 76.3 ^b^	2828.8 ± 648.6 ^b^	2727.1 ± 300.5 ^c^	496.3 ± 54.1 ^c^	359.6 ± 31.1 ^d^
3-ethyl-2-methyl-1,3-hexadiene	20.858	91	123.7 ± 33.9 ^a^	55.3 ± 2.0 ^a^	885.2 ± 89.3 ^b^	2642.0 ± 262.5 ^b^	2667.7 ± 503.6 ^c^	4082.4 ± 466.3 ^c^	nd	nd
2-Octenal	21.505	96	1579.5 ± 234.4	688.2 ± 10.8 ^a^	nd	nd	nd	4328.0 ± 485.5 ^b^	nd	1261.3 ± 197.0 ^c^
3-ethyl-2,5-dimethyl-pyrazine	22.057	95	755.6 ± 57.9 ^a^	33.2 ± 1.8 ^a^	2573.8 ± 295.7 ^b^	nd	1389.5 ± 76.7 ^c^	884.8 ± 87.4 ^b^	686.2 ± 170.0 ^a^	172.3 ± 6.3 ^c^
1-Octen-3-ol	22.551	90	1745.4 ± 50.9 ^a^	582.6 ± 1.8 ^a^	1159.0 ± 109.4 ^b^	14826.7 ± 91 ^b^	14411.6 ± 513.1 ^a,b^	22102.6 ± 83.4 ^c^	4870.2 ± 901.2 ^c^	5681.9 ± 378.1 ^d^
Furfural	23.161	94	402.7 ± 36.1 ^a^	106.1 ± 9.9 ^a^	13670.9 ± 906.9 ^b^	6002.6 ± 435 ^b^	3336.1 ± 585.2 ^c^	5524.3 ± 390.8 ^b^	2963.6 ± 251.6 ^c^	1940.0 ± 144.0 ^c^
1-(2-furanyl)-ethanone	24.754	91	nd	nd	nd	1432.3 ± 357.8 ^a^	nd	343.1 ± 82.6 ^b^	nd	nd
Trans-3-nonen-2-one	24.894	91	nd	nd	2387.5 ± 338.5 ^a^	1128.5 ± 144.8 ^a^	1703.4 ± 363.7 ^a^	2278.6 ± 401.3 ^b^	661.1 ± 82.2 ^b^	790.3 ± 87.9 ^c^
Benzaldehyde	25.375	96	249.6 ± 70.8 ^a^	63.9 ± 3.0 ^a^	1206.8 ± 139.5 ^b^	3596.6 ± 658.5 ^b^	1520.3 ± 149.3 ^c^	2338.7 ± 176.1 ^b^	920.0 ± 36.7 ^d^	530.3 ± 101.7 ^c^
6-Undecanone	25.531	94	nd	nd	nd	nd	nd	316.7 ± 52.8 ^a^	nd	215.9 ± 53.7 ^b^
Trans-2-Nonenal	25.838	90	nd	nd	1325.2 ± 37.7 ^a^	nd	779.1 ± 21.9 ^b^	nd	506.5 ± 60.0 ^c^	599.9 ± 43.9
1-Octanol	26.780	92	nd	nd	480.4 ± 44.3	nd	nd	905.7 ± 143.7	nd	nd
5-methyl-2-furfural	27.360	94	nd	nd	519.2 ± 40.7	3707.5 ± 620.9 ^a^	nd	1206.6 ± 269.9 ^b^	nd	514.1 ± 99.9 ^c^
Calarene	27.649	96	103.7 ± 26.1	18.1 ± 1.2	nd	nd	nd	nd	nd	nd
1-(2-pyridinyl)-ethanone	28.259	96	nd	nd	nd	546.6 ± 60.9 ^a^	238.8 ± 57.4	423.0 ± 75.3 ^a^	nd	nd
Furfuryl alcohol	30.251	95	nd	nd	462.1 ± 43.0 ^a^	7528.1 ± 832.1 ^a^	1007.8 ± 258.9 ^b^	2806.0 ± 165.8 ^b^	870.6 ± 73.0 ^c^	775.0 ± 98.2 ^c^
Myrtenol	33.660	96	80.0 ± 16.3	33.2 ± 2.5	nd	nd	nd	nd	nd	nd
Trans,cis-2,4-decadienal	34.126	96	166.9 ± 29.4	nd	nd	nd	nd	nd	nd	nd
Trans,trans-2,4-decadienal	34.128	96	nd	84.0 ± 3.7 ^a^	819.6 ± 106.1 ^a^	530.9 ± 58.9 ^b^	2388.3 ± 311.8 ^b^	1458.8 ± 138.3 ^c^	1750.4 ± 183.4 ^c^	3254.6 ± 621.1 ^d^
1-(1H-pyrrol-2-yl)-ethanone	37.819	94	nd	nd	nd	1423.8 ± 226.6 ^a^	1148.4 ± 136.4 ^a^	2044.3 ± 109.8 ^b^	225.6 ± 44.6 ^b^	126.9 ± 23.7 ^c^
1H-pyrrole-2-carboxaldehyde	38.983	95	nd	nd	nd	888.0 ± 122.5 ^a^	750.6 ± 67.3 ^a^	2041.6 ± 37.5 ^b^	684.1 ± 53.1 ^a^	275.8 ± 74.9 ^c^

t_R_ = Retention time (min). nd = Non-detected or detected with similarity < 90% with Wiley library. Q_f_: quality factor = % matching of the experimental mass spectra against those found in the Wiley database (Wiley7, Nist 05, J. Wiley & Sons Ltd., West Sussex, England). Different superscripts for each volatile compound within the same row and sunflower seed sample indicate statistically significant different values (*p* < 0.05).

## Data Availability

The data presented in this study are available on request from the corresponding author.

## References

[1] Food and Agriculture Organization of the United States FAOSTAT Database. http://www.fao.org/faostat/en/#data.

[2] CBI Product Factsheet: Sunflower Seeds in Europe.

[3] Salgın U., Döker O., Çalımlı A. (2006). Extraction of sunflower oil with supercritical CO2: Experiments and modeling. J. Supercrit. Fluids.

[4] Rai A., Mohanty B., Bhargava R. (2016). Supercritical extraction of sunflower oil: A central composite design for extraction variables. Food Chem..

[5] Chandrasekara N., Shahidi F. (2011). Oxidative Stability of Cashew Oils from Raw and Roasted Nuts. J. Am. Oil Chem. Soc..

[6] Roman O., Heyd B., Broyart B., Castillo R., Maillard M.N. (2013). Oxidative reactivity of unsaturated fatty acids from sunflower, high oleic sunflower and rapeseed oils subjected to heat treatment, under controlled conditions. LWT Food Sci. Technol..

[7] Pratt D.A., Tallman K.A., Porter N.A. (2011). Free Radical Oxidation of Polyunsaturated Lipids: New Mechanistic Insights and the Development of Peroxyl Radical Clocks. Acc. Chem. Res..

[8] Liu Z., Wei W., Huang G., Zhang W., Ni L. (2016). In vitro and in vivo evaluation of the prebiotic effect of raw and roasted almonds (*Prunus amygdalus*). J. Sci. Food Agric..

[9] Kong F., Singh R.P. (2009). Digestion of Raw and Roasted Almonds in Simulated Gastric Environment. Food Biophys..

[10] Sanchez-Bel P., Egea I., Pretel M., Flores F., Romojaro F., Martínez-Madrid M. (2011). Roasting and packaging in nitrogen atmosphere protect almond var. Guara against lipid oxidation. Food Sci. Technol. Int..

[11] Kita A., Figiel A. (2007). Effect of roasting on properties of walnuts. Pol. J. Food Nutr. Sci..

[12] Alasalvar C., Pelvan E., Topal B. (2010). Effects of roasting on oil and fatty acid composition of Turkish hazelnut varieties (*Corylus avellana* L.). Int. J. Food Sci. Nutr..

[13] Lin J.T., Liu S.C., Hu C.C., Shyu Y.S., Hsu C.Y., Yang D.J. (2016). Effects of roasting temperature and duration on fatty acid composition, phenolic composition, Maillard reaction degree and antioxidant attribute of almond (*Prunus dulcis*) kernel. Food Chem..

[14] Bolling B.W., Blumberg J.B., Chen C.-Y.O. (2010). The influence of roasting, pasteurisation, and storage on the polyphenol content and antioxidant capacity of California almond skins. Food Chem..

[15] Sanahuja A.B., Pérez S.E.M., Teruel N.G., García A.V., Moya M.S.P. (2021). Variability of Chemical Profile in Almonds (*Prunus dulcis*) of Different Cultivars and Origins. Foods.

[16] Juárez M.D., Osawa C.C., Acuña M.E., Sammán N., Gonçalves L.A.G. (2011). Degradation in soybean oil, sunflower oil and partially hydrogenated fats after food frying, monitored by conventional and unconventional methods. Food Control.

[17] Marmesat S., Velasco J., Ruiz-Méndez M.V., Dobarganes M.C. (2006). Oxidative quality of commercial fried nuts: Evaluation of a surface and an internal lipid fraction. Grasas Aceites.

[18] Aladedunye F., Przybylski R. (2013). Frying stability of high oleic sunflower oils as affected by composition of tocopherol isomers and linoleic acid content. Food Chem..

[19] Aladedunye F.A., Przybylski R. (2011). Frying Performance of Canola Oil Triacylglycerides as Affected by Vegetable Oils Minor Components. J. Am. Oil Chem. Soc..

[20] Beltrán A., PratS M.S., Maestre S.E., Grané N., Martín M.L. (2009). Classification of four almond cultivars using oil degradation parameters based on FTIR and GC data. J. Am. Oil Chem. Soc..

[21] Cherif A., Belkacemi K., Kallel H., Angers P., Arul J., Boukhchina S. (2009). Phytosterols, unsaturated fatty acid composition and accumulation in the almond kernel during harvesting period: Importance for development regulation. Comptes Rendus Biol..

[22] Valdés A., Beltrán A., Mellinas C., Jiménez A., Garrigós M.C. (2018). Analytical methods combined with multivariate analysis for authentication of animal and vegetable food products with high fat content. Trends Food Sci. Technol..

[23] Yaacoub R., Saliba R., Nsouli B., Khalaf G., Birlouez-Aragon I. (2008). Formation of Lipid Oxidation and Isomerization Products during Processing of Nuts and Sesame Seeds. J. Agric. Food Chem..

[24] Lim H.K., Tan C.P., Karim R., Ariffin A.A., Bakar J. (2010). Chemical composition and DSC thermal properties of two species of Hylocereus cacti seed oil: Hylocereus undatus and Hylocereus polyrhizus. Food Chem..

[25] Hao J., Xu X.-L., Jin F., Regenstein J.M., Wang F.-J. (2020). HS-SPME GC–MS characterization of volatiles in processed walnuts and their oxidative stability. J. Food Sci. Technol..

[26] Cordero C., Liberto E., Bicchi C., Rubiolo P., Schieberle P., Reichenbach S.E., Tao Q. (2010). Profiling food volatiles by comprehensive two-dimensional gas chromatography coupled with mass spectrometry: Advanced fingerprinting approaches for comparative analysis of the volatile fraction of roasted hazelnuts (*Corylus avellana* L.) from different origins. J. Chromatogr. A.

[27] Beltrán A., Grané N., Martín M., Garrigós M.C. (2011). Characterization of Almond Cultivars by the Use of Thermal Analysis Techniques. Application to Cultivar Authenticity. J. Am. Oil Chem. Soc..

[28] American Oil Chemists’ Society (1992). Official Methods and Recommended Practices of the American Oil Chemists’ Society.

[29] ISO (2017). ISO 3960:2017, Corrected Version 2009-05-15. Animal and Vegetable Fats and Oils—Determination of Peroxide Value—Iodometric (Visual) Endpoint Determination.

[30] IUPAC (1987). Determination of the p-Anisidine Value, Method 2.504. Standard Methods for the Analysis of Oils, Fats and Derivatives.

[31] Valdés A., Beltrán A., Karabagias I., Badeka A., Kontominas M.G., Garrigós M.C., Sanahuja A.B. (2015). Monitoring the oxidative stability and volatiles in blanched, roasted and fried almonds under normal and accelerated storage conditions by DSC, thermogravimetric analysis and ATR-FTIR. Eur. J. Lipid Sci. Technol..

[32] Li Q., Zhang H.-H., Claver I.P., Zhu K.-X., Peng W., Zhou H.-M. (2011). Effect of different cooking methods on the flavour constituents of mushroom (*Agaricus bisporus* (Lange) Sing) soup. Int. J. Food Sci. Technol..

[33] Mexis S.F., Badeka A.V., Riganakos K.A., Karakostas K.X., Kontominas M.G. (2009). Effect of packaging and storage conditions on quality of shelled walnuts. Food Control.

[34] Hassan F., Kaleem S., Ahmad M. (2011). Oil and fatty acid distribution in different circles of sunflower head. Food Chem..

[35] Gaca A., Kludská E., Hradecký J., Hajšlová J., Jeleń H.H. (2021). Changes in Volatile Compound Profiles in Cold-Pressed Oils Obtained from Various Seeds during Accelerated Storage. Molecules.

[36] Banas J., Maciejaszek I., Surowka K., Zawislak A. (2020). Temperature-induced storage quality changes in pumpkin and safflower cold-pressed oils. J. Food Meas. Charact..

[37] Neđeral S., Škevin D., Kraljić K., Obranović M., Papeša S., Bataljaku A. (2012). Chemical Composition and Oxidative Stability of Roasted and Cold Pressed Pumpkin Seed Oils. J. Am. Oil Chem. Soc..

[38] Silva L., Pinto J., Carrola J., Paiva-Martins F. (2010). Oxidative stability of olive oil after food processing and comparison with other vegetable oils. Food Chem..

[39] Anjum F., Anwar F., Jamil A., Iqbal M. (2006). Microwave roasting effects on the physico-chemical composition and oxidative stability of sunflower seed oil. J. Am. Oil Chem. Soc..

[40] Choe E., Min D.B. (2006). Mechanisms and Factors for Edible Oil Oxidation. Compr. Rev. Food Sci. Food Saf..

[41] Mexis S.F., Badeka A.V., Chouliara E., Riganakos K.A., Kontominas M.G. (2009). Effect of γ-irradiation on the physicochemical and sensory properties of raw unpeeled almond kernels (*Prunus dulcis*). Innov. Food Sci. Emerg. Technol..

[42] Casal S., Malheiro R., Sendas A., Oliveira B.P., Pereira J.A. (2010). Olive oil stability under deep-frying conditions. Food Chem. Toxicol..

[43] Poiana M.-A. (2012). Enhancing Oxidative Stability of Sunflower Oil during Convective and Microwave Heating Using Grape Seed Extract. Int. J. Mol. Sci..

[44] Velasco J., Dobarganes C. (2002). Oxidative stability of virgin olive oil. Eur. J. Lipid Sci. Tech..

[45] Man Y.B.C., Tan C.P. (2002). Comparative differential scanning calorimetric analysis of vegetable oils: II. Effects of cooling rate variation. Phytochem. Anal..

[46] Koontz J.L., Marcy J.E., O’Keefe S.F., Duncan S.E. (2009). Cyclodextrin inclusion complex formation and solid-state characterization of the natural antioxidants α-tocopherol and quercetin. J. Agric. Food Chem..

[47] Lodi A., Vodovotz Y. (2008). Physical properties and water state changes during storage in soy bread with and without almond. Food Chem..

[48] Perren R., Escher F., Harris L.J. (2013). Impact of roasting on nut quality. Improving the Safety and Quality of Nuts.

[49] Akil E., Castelo-Branco V.N., Costa A.M.M., Vendramini A.L.D.A., Calado V., Torres A.G. (2015). Oxidative Stability and Changes in Chemical Composition of Extra Virgin Olive Oils after Short-Term Deep-Frying of French Fries. J. Am. Oil Chem. Soc..

[50] Rovellini P., Fusari P., Venturini S. (2004). Seed oils: Nutritional quality and triglycerides profile. Riv. Ital. Delle Sostanze Grasse.

[51] Igoumenidis P.E., Konstanta M.A., Salta F.N., Karathanos V.T. (2011). Phytosterols in frying oils: Evaluation of their absorption in pre-fried potatoes and determination of their destruction kinetics after repeated deep and pan frying. Procedia Food Sci..

[52] Bendini A., Barbieri S., Valli E., Buchecker K., Canavari M., Toschi T.G. (2011). Quality evaluation of cold pressed sunflower oils by sensory and chemical analysis. Eur. J. Lipid Sci. Technol..

[53] Amor A.M.C.-D., Aguayo E., Collado-González J., Guy A., Galano J.-M., Durand T., Gil-Izquierdo Á. (2016). Impact of packaging atmosphere, storage and processing conditions on the generation of phytoprostanes as quality processing compounds in almond kernels. Food Chem..

[54] Ghazzawi H.A., Al-Ismail K. (2017). A Comprehensive Study on the Effect of Roasting and Frying on Fatty Acids Profiles and Antioxidant Capacity of Almonds, Pine, Cashew, and Pistachio. J. Food Qual..

[55] Aceña L., Vera L., Guasch J., Busto O., Mestres M. (2011). Determination of Roasted Pistachio (*Pistacia vera* L.) Key Odorants by Headspace Solid-Phase Microextraction and Gas Chromatography−Olfactometry. J. Agric. Food Chem..

[56] Guillen M.D., Goicoechea E. (2007). Detection of Primary and Secondary Oxidation Products by Fourier Transform Infrared Spectroscopy (FTIR) and1H Nuclear Magnetic Resonance (NMR) in Sunflower Oil during Storage. J. Agric. Food Chem..

